# Antifungal Activity against *Botryosphaeriaceae* Fungi of the Hydro-Methanolic Extract of *Silybum marianum* Capitula Conjugated with Stevioside

**DOI:** 10.3390/plants10071363

**Published:** 2021-07-03

**Authors:** Natalia Langa-Lomba, Laura Buzón-Durán, Eva Sánchez-Hernández, Pablo Martín-Ramos, José Casanova-Gascón, Jesús Martín-Gil, Vicente González-García

**Affiliations:** 1 Instituto Universitario de Investigación en Ciencias Ambientales de Aragón (IUCA), EPS, Universidad de Zaragoza, Carretera de Cuarte, s/n, 22071 Huesca, Spain; natalialangalomba@gmail.com (N.L.-L.); jcasan@unizar.es (J.C.-G.); 2 Plant Protection Unit, Agrifood Research and Technology Centre of Aragón, Instituto Agroalimentario de Aragón—IA2 (CITA-Universidad de Zaragoza), Avda. Montañana 930, 50059 Zaragoza, Spain; vgonzalezg@aragon.es; 3 Agriculture and Forestry Engineering Department, ETSIIAA, Universidad de Valladolid, Avenida de Madrid 44, 34004 Palencia, Spain; laura.buzon@uva.es (L.B.-D.); eva.sanchez.hernandez@uva.es (E.S.-H.); jesus.martin.gil@uva.es (J.M.-G.)

**Keywords:** coniferyl alcohol, ferulic acid, grapevine trunk diseases, milk thistle, stevioside

## Abstract

*Silybum marianum* (L.) Gaertn, viz. milk thistle, has been the focus of research efforts in the past few years, albeit almost exclusively restricted to the medicinal properties of its fruits (achenes). Given that other milk thistle plant organs and tissues have been scarcely investigated for the presence of bioactive compounds, in this study, we present a phytochemical analysis of the extracts of *S. marianum* capitula during the flowering phenological stage (stage 67). Gas chromatography–mass spectroscopy results evidenced the presence of high contents of coniferyl alcohol (47.4%), and secondarily of ferulic acid ester, opening a new valorization strategy of this plant based on the former high-added-value component. Moreover, the application of the hydro-methanolic extracts as an antifungal agent has been also explored. Specifically, their activity against three fungal species responsible for the so-called *Botryosphaeria* dieback of grapevine (*Neofusicoccum parvum*, *Dothiorella viticola* and *Diplodia seriata*) has been assayed both in vitro and in vivo. From the mycelial growth inhibition assays, the best results (EC_90_ values of 303, 366, and 355 μg·mL^−1^ for *N. parvum*, *D. viticola*, and *D. seriata*, respectively) were not obtained for the hydroalcoholic extract alone, but after its conjugation with stevioside, which resulted in a strong synergistic behavior. Greenhouse experiments confirmed the efficacy of the conjugated complexes, pointing to the potential of the combination of milk thistle extracts with stevioside as a promising plant protection product in organic Viticulture.

## 1. Introduction

*Silybum marianum* (L.) Gaertn (syn. *Carduus marianus* L.), commonly known as milk thistle, St. Mary’s Thistle, or wild artichoke, is an herbaceous plant of the *Asteraceae* family. Native to the Mediterranean area, it is nowadays grown in many countries as a medicinal plant, due to the variety of biological activities—mostly linked to the hepatoprotective properties and anti-carcinogenic capacity—associated with the main pharmacological active ingredient extracted from its achenes (fruits): silymarin [1,2].

The standardized extract obtained from the dried fruits of *S. marianum* contains 70–80% of silymarin and 20–30% of polymeric and oxidized polyphenolic compounds [3]. Silymarin is a flavonolignan complex of polyphenolic molecules, which includes diasterereoisomers silybin A and silybin B (whose mixture in a 1:1 ratio is named silibinin), silydianin, silychristin, isosilychristin, isosilybin A and isosilybin B, and the taxifolin flavanonol [4]. Biosynthesis of silybins from taxifolin and coniferyl alcohol is schematized in Figure S1 [5].

Most research has been focused on the study of silymarin, or its major compound silybin, instead of the plant as a whole. The concentration of silymarin is organ-dependent, and it is only localized in the outer portion of the fruit, which includes all the cell layers from the pericarp epidermis to the albumen, and embryos [6], accounting for 1.5–4.3% of the fruit weight [7]. Silymarin is not present in flowers, stems, or leaves, and it is not found in steps before the development of fruit [8,9], which explains why other milk thistle plant organs have been scarcely investigated for bioactive compounds: total polyphenol and flavonoid contents in leaves’ extracts were studied by Saidi, et al. [10]; a phytochemical screening and gas chromatography–mass spectrometry (GC–MS) analysis of bioactive compounds present in ethanolic leaves extract was conducted by Mani, et al. [11]; and Sulas, et al. [12] studied the concentrations of crude protein, fat, total phenolics, and total flavonoids in leaves, heads, and stems.

A thorough search of the relevant literature yielded no analyses of the phytochemicals present in the capitula in the flowering stage, prior to seed maturation. Nonetheless, the existence of some precursors proposed in the bibliography, such as coniferyl alcohol or ferulic acid, may be expected (Figure S2) [5]. Coniferyl alcohol is one of the main monolignols of angiosperm dicotyledons [13], and it is distributed throughout the milk thistle plant [8]. It is associated with the defense mechanisms of trees and is known to have inhibitory activity against the growth of fungi [14,15]. Ferulic acid (4-hydroxy-3-methoxycinnamic acid) and its precursors, *p*-coumaric acid, and caffeic acid, are metabolites in the biosynthesis of lignins. These compounds are intermediates in the biosynthesis of some important natural products very often found in plants, such as *p*-coumaryl alcohol, curcumin, chlorogenic acid, diferulic acids, sinapic acid, synapyl alcohol, coniferyl alcohol, vanillin, etc. [16].

Regarding the antifungal activity of the above-cited compounds, the literature indicates that silymarin is effective against yeasts like *Candida* spp. (*C. albicans* (C.P. Robin) Berkhout, *C. krusei* (Castellani) Berkhout, and *C. glabrata* (H.W. Anderson) S.A. Mey. & Yarrow) [17,18], and that coniferyl derivatives are effective against *Cladosporium cucumerinum* Ellis & Arthur and *C. albicans* [19]. Ferulic acid has been reported as an inhibitor of the fungal growth of, for instance, *Pythium* spp. [20], *Fusarium* spp. [21,22], and *Aspergillus* spp. [23,24]. Esters of ferulic acid were found to be more potent antimicrobial agents than amides and anilides, according to Khatkar, et al. [25], and their high antimicrobial activity was evidenced by the results of Mahiwal, et al. [26].

Concerning the control of Botryosphaeriaceous fungi—which are recognized as aggressive plant pathogens on different types of hosts, from agricultural crops to ornamental and forest species—[27], ferulic acid has been assayed against taxa like *Diplodia seriata* and *Neofusicoccum parvum*, and against other grapevine trunk pathogens such as *Eutypa lata* (Pers.) Tul. & C.Tul., *Phaeomoniella chlamydospora* (W. Gams, Crous, M.J. Wingf. & Mugnai) Crous & W. Gams and *Phaeoacremonium minimum* (Tul. & C. Tul.) Gramaje, L. Mostert & Crous [28,29,30], but the activity of *S. marianum* extracts or coniferyl alcohol has not been assayed to date, in spite of the importance of these phytopathogens in economically important crops like Viticulture [31].

In this study, a phytochemical analysis of the extracts of *S. marianum* capitula during the flowering phenological stage (stage 67, when the head disk is covered by open florets (i.e., during the flowering stage and before the development of fruit)) is presented, with the aim of exploring the presence of high-added-value components and the potential application the hydro-methanolic extracts as antifungal agents against three *Botryosphaeriaceae* species that play a major role in the so-called grapevine trunk diseases (GTDs). To circumvent the bioavailability problems associated with the very low solubility in water of ferulic acid [32], coniferyl alcohol, and other constituents, inclusion compounds or conjugate complexes with terpene glycosides may be formed [33]. In this study, stevioside (the major constituent of *Stevia rebaudiana* (Bertoni) Bertoni extract), which has antifungal properties, has been chosen to form such conjugate complexes, aiming at an enhancement of activity through synergism.

## 2. Material and Methods

### 2.1. Plant Material, Reagents, and Fungal Isolates

The specimens of *S. marianum* under study were collected in the banks of Carrión river as it passes through the town of Palencia (Spain) during stage 67 (or 6N7) according to the extended BBCH scale [34]. This stage was chosen because silybins precursors (Figure S1) should not have yet been consumed. The capitula of *S. marianum* were shade-dried and pulverized to fine powder in a mechanical grinder. Different specimens (*n* = 25) were thoroughly mixed to obtain a composite sample.

Chitosan (CAS 9012-76-4; high MW: 310,000–375,000 Da) was supplied by Hangzhou Simit Chem. and Tech. Co. (Hangzhou, China). Neutrase^TM^ 0.8 L enzyme was supplied by Novozymes A/S (Bagsværd, Denmark). Stevioside (CAS 57817-89-7, 99%) was purchased from Wako Chemicals GmbH (Neuss, Germany). Coniferyl alcohol (CAS 458-35-5, 98%), ferulic acid (CAS 537-98-4, European Pharmacopoeia Reference Standard), sodium alginate (CAS 9005-38-3), calcium carbonate (CAS 471-34-1, ≥99.0%), and methanol (CAS 67-56-1, UHPLC, suitable for mass spectrometry) were acquired from Sigma-Aldrich Química (Madrid, Spain). PDA (potato dextrose agar) was supplied by Becton Dickinson (Bergen County, NJ, USA).

The three fungal pathogens (Table 1) were supplied as lyophilized vials (later reconstituted and refreshed as PDA subcultures) by the Agricultural Technological Institute of Castilla and Leon (ITACYL, Valladolid, Spain) [35].

### 2.2. Preparation of Plant Extracts

*Silybum marianum* capitula samples were mixed (1:20, *w*/*v*) with a methanol/water solution (1:1 *v*/*v*) and heated in a water bath at 50 °C for 30 min, followed by sonication for 5 min in pulse mode with a 1 min stop for each 2.5 min, using a probe-type ultrasonicator model UIP1000hdT (Hielscher Ultrasonics, Teltow, Germany; 1000 W, 20 kHz). The solution was then centrifuged at 9000 rpm for 15 min and the supernatant was filtered through Whatman No. 1 paper. Finally, aliquots of the extract were lyophilized for infrared spectroscopy analyses.

### 2.3. Physicochemical Characterization of S. marianum Extracts

The infrared vibrational spectra were registered using a Thermo Scientific (Waltham, MA, USA) Nicolet iS50 Fourier-transform infrared spectrometer, equipped with an in-built diamond attenuated total reflection (ATR) system. The spectra were collected with a 1 cm^−1^ spectral resolution over the 400–4000 cm^−1^ range, taking the interferograms that resulted from co-adding 64 scans. The spectra were then corrected using the advanced ATR correction algorithm [36] available in OMNIC^TM^ software suite.

The hydroalcoholic plant extracts were studied by gas chromatography–mass spectrometry (GC–MS) at the Research Support Services (STI) at Universidad de Alicante (Alicante, Spain), using a gas chromatograph model 7890A coupled to a quadrupole mass spectrometer model 5975C (both from Agilent Technologies). The chromatographic conditions were: injection volume = 1 µL; injector temperature = 280 °C, in splitless mode; initial oven temperature = 60 °C, 2 min, followed by ramp of 10 °C/min up to a final temperature of 300 °C, 15 min. The chromatographic column used for the separation of the compounds was an Agilent Technologies HP-5MS UI of 30 m length, 0.250 mm diameter and 0.25 µm film. The mass spectrometer conditions were: temperature of the electron impact source of the mass spectrometer = 230 °C and of the quadrupole = 150 °C; ionization energy = 70 eV. NIST11 library and Adams [37] were used for compound identification.

### 2.4. Preparation of Bioactive Formulations

The stevioside–*S. marianum*, stevioside–coniferyl alcohol, and stevioside–ferulic acid conjugate complexes were obtained by mixing of the respective solutions in a 1:1 (*v/v*) ratio. The mixture was then sonicated for 15 min in five 3-min periods (so that the temperature did not exceed 60 °C) using a probe-type ultrasonicator [38].

### 2.5. Antifungal Activity Assessment

#### 2.5.1. In Vitro Tests of Mycelial Growth Inhibition

The antifungal activity of the different treatments was determined using the agar dilution method according to EUCAST standard antifungal susceptibility testing procedures [39], by incorporating aliquots of stock solutions onto the PDA medium to obtain concentrations in the 62.5–1500 μg·mL^−1^ range. The solutions were added to the PDA after being sterilized in an autoclave, when the temperature of the medium was close to that of polymerization (over 60 °C), in the same way that antibiotics are usually incorporated into these synthetic media. Mycelial plugs (⌀ = 5 mm), from the margin of 1-week-old PDA cultures of *N. parvum*, *D. viticola* or *D. seriata*, were transferred to the center of plates incorporating the above-mentioned concentrations for each treatment (3 plates per treatment/concentration, with 2 replicates). Plates were then incubated at 25 °C in the dark for a week. PDA medium without any amendment was used as control. Mycelial growth inhibition was estimated according to the formula: ((*d_c_* − *d_t_*)/*d_c_*) × 100, where *d*_c_ and *d*_t_ represent the average diameters of the fungal colony of the control and of the treated fungal colony, respectively. Effective concentrations (EC_50_ and EC_90_) were estimated using PROBIT analysis in IBM SPSS Statistics v.25 (IBM; Armonk, NY, USA) software. The level of interaction, i.e., synergy factors, were determined according to Wadley’s method [40].

#### 2.5.2. Greenhouse Bioassays on Grafted Plants

Together with the experiments of mycelial growth inhibition in vitro, bioassays with stevioside–*S. marianum* conjugate complexes were performed in living grapevine plants in order to scale the protective capabilities of these compounds against certain selected *Botryosphaeriaceae* species usually associated with GTD symptoms on young grapevine plants. Especially, *N. parvum*, *D. viticola*, and *D. seriata* were selected for the in vivo assay due to their significant presence as part of the contingent of fungi associated with decay problems in young vine plants [41] in Spain and other viticultural areas internationally. Plant material consisted of 30 plants each of cultivars ‘Tempranillo’ (CL. 32 clone) (2-years old) and ‘Garnacha’ (VCR3 clone) (one year old) grafted on 775P and 110R rootstocks, respectively (60 plants in total). Each grapevine plant was grown on a 3.5 L plastic pot containing a mixed substrate of moss peat and sterilized natural soil (75:25), incorporating slow release fertilizer when needed. Plants were kept in the greenhouse with drip irrigation and anti-weed ground cover for six months (June–December 2020). One week after placing them in the pots, grapevine plants were artificially inoculated with the mentioned three *Botryosphaeriaceae* taxa along with the stevioside–*S. marianum* treatment. Five repetitions were arranged for each pathogen/control product combination and variety (‘Tempranillo’ and ‘Garnacha’), together with 8 positive controls (4 per grapevine variety) inoculated only with the pathogens, plus 6 negative controls (incorporating only the bioactive product), 3 for each variety (Table S1). Artificial inoculations of the pathogens and the control product were carried out directly on the trunk of the living plants at two sites per plant stand (separated a minimum of 5 cm among them) below the grafting point and not reaching the root crown. For the pathogens, agar plugs coming from 5-days-old fresh PDA cultures of each species were used as fungal inoculum. In the mentioned two inoculation points of each grapevine plant, slits (made up with a scalpel) of approx. 15 mm in diameter and 5 mm deep were done. After this, 5 mm diameter agar plugs were inoculated in contact with vascular tissues in the stem. Calcium alginate beads served as a dispersal matrix for the different control products and conjugates assayed, and were placed at both sides of the agar plug. For this, beads were prepared as follows: the control product was added to a 3% sodium alginate solution in a 2:8 ratio (20 mL compound/80 mL sodium alginate). Then, this solution was dispensed drop by drop onto a 3% calcium carbonate solution resulting in beads of 4–6 mm diameter containing the different control treatments. Finally, both agar plugs and beads were covered with cotton soaked in sterile bi-distilled water and sealed with Parafilm^TM^ tape. During the assay period, application of copper to control powdery mildew outbreaks was performed in mid-July, together with a first sprouting (followed by periodic sprouting). Grapevine plants were visually examined weekly during the whole assay period, and the presence of foliar symptoms—including both internerval and nerval necroses—was scored to establish correlations between these and vascular symptoms at the end of bioassay. After six months in the greenhouse, plants were removed and two sections of the inoculated stems between the grafting point and the root crown were prepared, sectioned longitudinally, and the length of the vascular necroses caused by the different pathogens evaluated. Thus, the length of the vascular necroses was measured longitudinally on upper and lower directions from the inoculation point for both halves of the longitudinal cut, and the averages were statistically analyzed and compared depending on the type of pathogen and product formulation employed. All the data were compared with controls. Finally, grapevine plants removed and measured at the end of the assay were also processed (after taking measures) to re-isolate the different pathogens previously inoculated. Then, wood chips (0.5 cm long) exhibiting vascular necroses (1–2 cm around the wounds) were washed, surface sterilized, placed on PDA plates amended with streptomycin sulphate (to prevent bacterial contamination) and incubated at 26 °C in the dark in a culture chamber for 2–3 days to fulfil Koch’s postulates.

### 2.6. Statistical Analyses

The results of the in vitro inhibition of mycelial growth of the three phytopathogens by the different concentrations of the treatments were statistically analyzed using one-way analysis of variance (ANOVA), followed by post hoc comparison of means through Tukey’s test at *p* < 0.05 (provided that the homogeneity and homoscedasticity requirements were satisfied, according to the Shapiro–Wilk and Levene tests). In the case of the greenhouse assay results, since the normality and homoscedasticity requirements were not met, Kruskal–Wallis non-parametric test was used instead, with Conover–Iman test for post hoc multiple pairwise comparisons. R statistical software was used [42].

## 3. Results

### 3.1. Vibrational Characterization

The assignments of the major absorption IR bands in *S. marianum* extract spectrum (Figure S3) are presented in Table 2. The most prominent bands occurred at 3335, 1651–1602, 1457, 1313, 1242, and 1029 cm^−1^. The band at 3335 cm^−1^ is attributed to phenolic (OH) vibrations; the multi-peak band at 1651 cm^−1^ to mixed (C=O) amide and (C=C) vibrations; the peak at 1515 cm^−1^ (typical of ferulic acid and vanillin) to >C=C< aromatic; the peak at 1457 cm^−1^ to symmetric aromatic ring stretching vibration (C=C ring); and the peaks at 1030 cm^−1^ and 779 cm^−1^ to C-O stretching and C=C, respectively (both vanillin-related peaks).

### 3.2. Gas Chromatography–Mass Spectrometry (GC-MS)

GC-MS analyses of the hydro-methanolic extract of *S. marianum* (Figure S4) allowed for the identification of 4-((1E)-3-hydroxy-1-propenyl)-2-methoxyphenol (also named coniferyl alcohol or *γ*-hydroxyisoeugenol); its analogue *trans*-isoeugenol; 2-propenoic acid, 3-(4-hydroxy-3-methoxyphenyl)-, methyl ester (known as ferulic acid methyl ester); 2-methoxyphenol; and 4-hydroxy-3-methylacetophenone as the main components (Table 3, Figure S5).

### 3.3. Antifungal Activity

#### 3.3.1. In Vitro Growth Inhibition Tests

**Figure 1:**
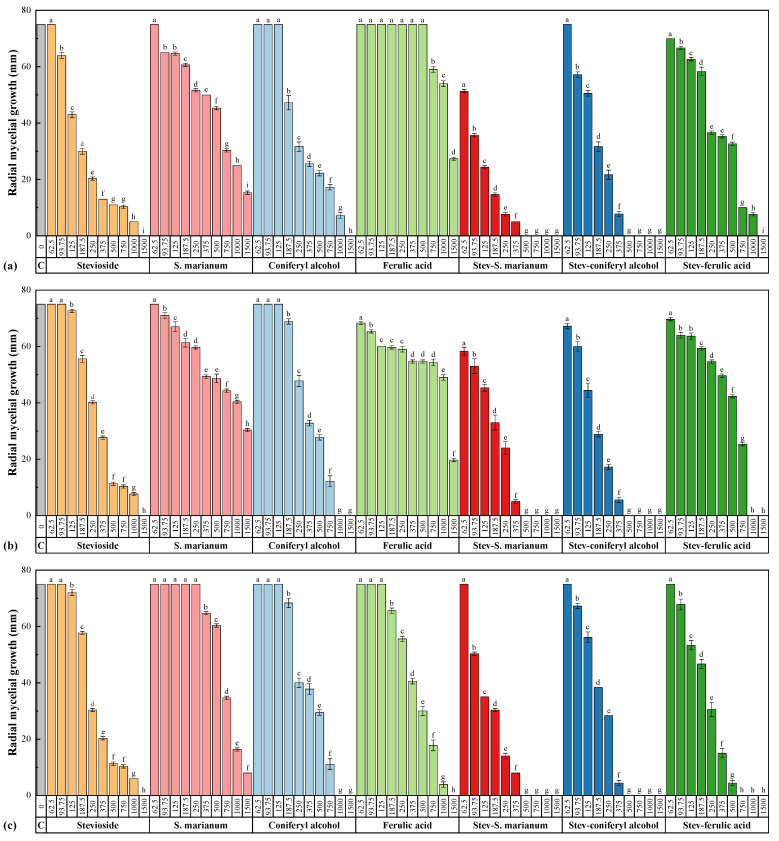
Colony growth measures of (**a**) *N. parvum*, (**b**) *D. viticola*, and (**c**) *D. seriata* strains when cultured in PDA plates containing the various control products (viz. stevioside, *S. marianum* hydromethanolic extract, coniferyl alcohol, ferulic acid, stevioside–*S. marianum*, stevioside–coniferyl alcohol and stevioside–ferulic acid conjugate complexes) at concentrations in the 62.5–1500 μg·mL^−1^ range. The same letters above concentrations indicate that they are not significantly different at *p* < 0.05. Error bars represent standard deviations.

The results of the mycelial growth inhibition tests are summarized in Figure 1. When tested alone, a higher efficacy of coniferyl alcohol as compared to ferulic acid could be observed: full inhibition was only reached for the former in the case of *N. parvum* and *D. viticola*. In the case of *D. seriata*, for which both treatments resulted in full inhibition, it was attained at a lower dose for coniferyl alcohol (1000 vs. 1500 μg·mL^−1^). Hence, the antifungal efficacy found for the extracts should be mostly ascribed to its main constituent. On the other hand, upon conjugation with stevioside, a clear enhancement in terms of efficacy was found in all cases, which was particularly evident in the case of the extracts, for which even higher inhibition than that of the coniferyl alcohol conjugates was attained at almost all concentrations against the three pathogens.

If effective concentrations are compared (Table 4), differences in the efficacy of the treatments as a function of the pathogen could be observed for some of the treatments more clearly: for instance, a slightly higher efficacy of stevioside and stevioside–*S. marianum* conjugate complex was found against *N. parvum*, and *D. seriata* seemed to be particularly sensitive to ferulic acid and its conjugate. On the other hand, the response of the three fungi to the coniferyl alcohol-based treatments was very similar.

In concordance with the above statements, the calculation of synergy factors (Table 5) indicated a strong synergistic behavior for the stevioside–*S. marianum* conjugate, with SF values in the 2.7–5.1 range.

#### 3.3.2. Greenhouse Bioassays

Protective tests conducted on grafted plants with the stevioside–milk thistle treatment confirmed its efficacy in more realistic conditions (i.e., closer to field ones): the application of the conjugate complex resulted in statistically significant differences as compared to the positive (pathogen) controls in all cases (Table 6). It is worth noting that the median lengths of the vascular necroses were higher in the case of treated plants artificially inoculated with *N. parvum* than for treated plants artificially inoculated with the other two taxa (for which the effectiveness would be similar), which may be regarded as an unexpected result, provided that the associated EC_90_ value was the lowest in the in vitro tests. This point was confirmed by including the fungus taxon in the statistical analysis as a second independent variable (Table S2). However, no statistically significant differences were observed among the three fungi in terms of necrosis lengths in the positive controls. Interestingly, in the two-factor analysis, the lengths of the necroses for the treated plants artificially inoculated with *D. viticola* were not significantly different from those of the negative controls, pointing to a particularly high inhibition of this pathogen.

## 4. Discussion

### 4.1. Valorization of Coniferyl Alcohol and Ferulic Acid

As expected from the phenological stage in which the plants were collected (flowering, before fruit ripening) and taking into consideration that the entire capitula were used for the extraction (not only the fruits), the panel of extracted components was different from those present in the commercially available milk thistle seed extract: instead of silybin (A and B) and isosilybin (A and B), coniferyl alcohol and other eugenol analogues were identified; and instead of vanillin, the quantitative presence of its precursor (ferulic acid methyl ester) was evidenced.

Coniferyl alcohol is a valuable chemical, which reaches 350 USD·g^−1^ when bought from commercial suppliers such as Sigma-Aldrich. Current approaches to obtain coniferyl alcohol are either inefficient, harmful (*Penicillium simplicissimum* (Oudemans) Thom vanillyl alcohol oxidase (PsVAO) can be used to produce it, but it intrinsically produces harmful byproduct H_2_O_2_), or expensive (its synthesis involves expensive substrates and catalyst and harsh reaction conditions) [43,44]. These limitations can be overcome with the ultrasonic-assisted hydro-methanolic extraction of the capitula, reported in this paper, which may allow for the obtainment of the phenylpropanoid coniferyl alcohol with a yield of 50–80%. Alternative extractive approaches, such as the use of ionic liquid analogs (deep eutectic solvents) as extractive solvents [45], microwave-assisted extraction, dynamic maceration process [46], negative pressure cavitation-assisted extraction with macroporous resin enrichment [47], etc., should nonetheless be explored in order to optimize the yield.

**Figure 2:**
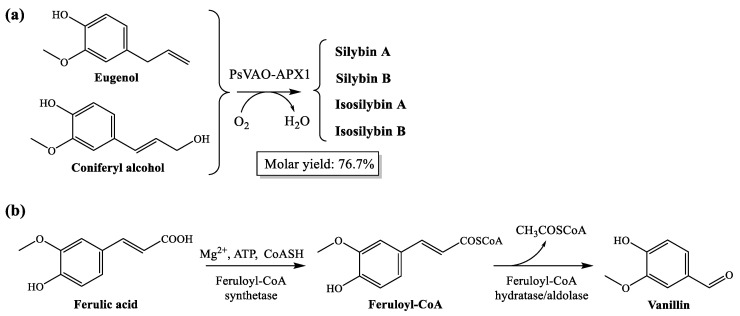
(**a**) Highly efficient enzymatic cascade engineered for biotransformation of eugenol to silybin and isosilybin. Adapted from [44]. (**b**) Schematic representation of the non-β-oxidative pathway for conversion of ferulic acid into vanillin, according to [51].

In the case that the production of silymarin-based drugs is desired, the biotransformation of eugenol and coniferyl alcohol to silybin and isosilybin can be efficiently attained by the oxidation of the precursors by milk thistle ascorbate peroxidase (APX1), as shown in Figure 2a.

In the same way, the finding of a 10:1 ratio for the ferulic acid–vanillin pair confirms that, for *S. marianum* capitula during the flowering phenological stage in a hydro-methanolic medium, the presence of the ferulic acid precursor is enhanced. Should vanillin be the desired chemical to obtain, the quantitative conversion of ferulic acid into vanillic acid could be feasible in presence of *Pseudomonas* spp. [48] (Figure 2b). The polypore species *Pycnoporus cinnabarinus* (Jacq.) P. Karst. has also been proposed for the production of vanillin from ferulic acid [49], although the vanillin produced is either rapidly converted to other products or utilized by the fungus as a source of carbon and energy. Genetic engineering has been applied to produce vanillin from ferulic acid using metabolically engineered *Escherichia coli* (Migula, 1895) Castellani and Chalmers, 1919 [50,51]. Another alternative would be the use of packed bed-stirred fermenters using *Bacillus subtilis* (Ehrenberg, 1835) Cohn, 1872 [52].

It should be noted that the extraction of coniferyl alcohol and ferulic acid would not preclude the valorization of the rest of the biomass as a feedstock for bioenergy production [53,54,55].

### 4.2. Efficacy of the Treatments

Stevioside, a terpene glycoside obtained from *Stevia rebaudiana* (Bertoni) Bertoni, showed a high inhibitory activity, comparable to that of coniferyl alcohol. Since—to the best of the authors’ knowledge—this is the first time that this compound is assayed against *Botryosphaeriaceae* fungi, no comparisons with similar taxa in terms of MIC values are available. However, the detected antifungal activity would be in good agreement with the results presented by other authors, who reported an inhibitory effect against other fungi (*Alternaria solani* Sorauer, *Helminthosporium solani* Durieu & Montagne*, Aspergillus* spp.*, Fusarium* spp., *Penicillium chrysogenum* Thom, or *Botrytis cinerea* Pers., among others) [56,57,58,59,60], with MIC values varying over a wide range (from 250 to 3000 μg·mL^−1^).

With regard to the activity of *S. marianum*-derived phytochemicals, the antifungal activity of silymarin/silibinin against *Candida* spp. and its underlying mechanism has been studied by Yun and Lee [18,61] and Janeczko and Kochanowicz [62]. Fernández, et al. [63] found significant inhibition against *Fusarium graminearum* Schwabe for four flower defensins from milk thistle. Safarpoor, et al. [64] reported moderate antifungal activities of ethanolic extracts of milk thistle against *C. albicans* and *Aspergillus oryzae* (Ahlb.) Cohn. Some antifungal activity was also reported for leaf and flower ethanolic extracts by Keskin, et al. [65] against *C. albicans*. Nonetheless, in these two latter studies no details were provided about the phenological stage in which the plants were collected, and effective concentrations were not reported.

Concerning the antifungal action of coniferyl alcohol, no data against GTD-related fungi is available in the literature, but—according to Kuc [66]—it has strong antifungal properties. For instance, coniferyl alcohol and its derivatives have been shown to be effective against *Colletotrichum lagenarium* (Pass.) Ellis & Halst., *C. cucumerinum*, *Melampsora lini* Ehrenb.) Lév., and *C. albicans* [19,67,68].

In relation to ferulic acid, it has been assayed against GTDs, and, according to Lambert, et al. [28], it is the phenolic acid with the strongest activity against *D. seriata, N. parvum, E. lata*, and *P. chlamydospora*. The same group, in a different study, found inhibition percentages in the 23–35% range for ferulic acid at a concentration of 500 μM (97 μg·mL^−1^) against different *N. parvum* isolates [69]. Gómez, et al. [29] reported half maximal effective concentrations of 3530 and 4740 μg·mL^−1^ against *Botryosphaeriaceae* sp. and *P. minimum*, and Dekker, et al. [30] found an EC_50_ value of 15 mM (2913 μg·mL^−1^) against *Botryosphaeria* sp. Srivastava, et al. [70] found that ferulic acid at 25 mM (4855 μg·mL^−1^) resulted in ca. 80% and ca. 70% mycelium growth inhibition of *B. rhodina* (Berk. & M.A. Curtis) Arx and *B. ribis* Grossenbacher & Duggar, respectively; and 100% inhibition was attained at 20 mM (3885 μg·mL^−1^) in the case of *B. obtusa* (Schwein.) Shoemaker. Such concentrations are close to the EC_90_ values against *N. parvum* and *D. viticola* reported in this work.

Regarding the conjugate complexes with stevioside, no data is available against GTDs. The most similar assayed product would be the stevioside:silymarin conjugate complexes (in a 1:1 molar ratio) tested against *Fusarium culmorum* (Wm.G. Sm.) Sacc., for which an EC_90_ value of 160 µg·mL^−1^ and a synergy factor of 1.43 were reported [71]. No antifungal efficacy data is available for stevioside–coniferyl alcohol conjugate complexes, but stevioside–ferulic acid inclusion compounds (with different molar ratios to the one assayed herein, and involving a more complex preparation procedure) have been tested against *F. culmorum* and *Phytophthora cinnamomi* de Bary. In the former case, composites based on stevioside:ferulic acid inclusion compounds (in a 5:1 molar ratio), combined with chitosan oligomers in hydroalcoholic solution or in choline chloride:urea deep eutectic solvent media, led to EC_90_ values in the 377–713 µg·mL^−1^ range against *F. culmorum* [72], depending on the dispersion medium. In the case of *P. cinnamomi*, inclusion compounds from stevioside and ferulic acid in 6:1 ratio, dispersed in a hydroalcoholic solution of chitosan oligomers, resulted in EC_90_ values of 446–450 µg·mL^−1^ (depending on the presence/absence of silver nanoparticles) [73,74].

## 5. Conclusions

In the hydromethanolic extract of *Silybum marianum* capitula, during the flowering stage, high contents of coniferyl alcohol derivatives and ferulic acid esters were found, instead of other chemical species such as the silymarin complex or vanillin. Given the high price of coniferyl alcohol, this may pose an alternative valorization strategy for this weed, compatible with a subsequent valorization for bioenergy purposes. Concerning the antifungal activity of the hydroalcoholic extract, the EC_50_ and EC_90_ values obtained against the three studied Botryospheriaceous grapevine pathogens (*N. parvum, D. viticola* and *D. seriata*) were in the 557–1088 and 1461–9942 μg·mL^−1^ range, respectively. However, a significant efficacy enhancement (with EC_50_ and EC_90_ values in the 87–148 and 303–596 μg·mL^−1^, respectively) was obtained by formation of conjugate complexes of the hydrometanolic extract of *S. marianum* with stevioside, evidencing a clear synergistic behavior (with synergy factor values of up to 5.1) as a result of the solubility and bioavailabity improvement. The efficacy of the stevioside–milk thistle conjugate complexes was further assessed in artificially inoculated grafted plants, obtaining significant differences in the vascular necroses lengths vs. the positive controls in all cases. The presented results support the possibility of extending the applications of milk thistle to agriculture as an antifungal agent, in particular for the protection of grapevines against certain fungal trunk diseases.

## Figures and Tables

**Table 1:** Fungal isolates used in the study.

Code	Isolate	Binomial Nomenclature	Geographical Origin	Host/Date
ITACYL_F111	Y-091-03-01c	*Neofusicoccum parvum* (Pennycook & Samuels) Crous, Slippers & A.J.L.Phillips	Spain (Navarra, nursery)	Grapevine (‘Verdejo’) 2006
ITACYL_F118	Y-103-08-01	*Dothiorella viticola* A.J.L.Phillips & J.Luque	Spain (Extremadura)	Grapevine 2004
ITACYL_F098	Y-084-01-01a	*Diplodia seriata* De Not.	Spain (DO Toro)	Grapevine (‘Tempranillo’) 2004

**Table 2:** Main bands in the FTIR spectra of *S. marianum* lyophilized hydromethanolic extract, silymarin and ferulic acid. Band positions are expressed in cm^−1^.

*Silybum marianum*	Silymarin	Ferulic Acid	Assignments
3335		3331	OH group in phenolic compounds
	3279		
3069			
2918	2932	2926	O–H stretching
1651 1634		1649	skeletal vibration due to aromatic C=C ring stretching and C=O stretching
1602		1605	aromatic C=C stretching
1558			>C=C< aromatic
1515 1457	1458	1510	symmetric aromatic ring stretching vibration (C=C ring)
1429	1434		olefinic C–H
1313		1329	C–H vibration of the methyl group
		1275	Carboxylic acid C=O stretching
1242	1257		
	1126		in plane =C–H bending/C=C stretching
1030	1076		C–O stretching/O-H out plane bending
	941		
779	721	693	C=C on the aromatic ring methylene rocking vibration

**Table 3:** Phytochemical compounds identified by GC-MS in the hydromethanolic extract of *S. marianum* capitula in phenological stage 67.

Peak	Rt (min)	Area (%)	Tentative Assignments
1	4.8755	2.67	methoxy-phenyl-oxime
2	6.0099	3.50	glycerin
3	7.3293	2.62	hexamethyl-cyclotrisiloxane; tris(tert-butyldimethylsilyloxy)arsane
4	7.6360	7.31	2-methoxy-phenol
5	9.4764	2.48	2,3-dihydro-benzofurane
6	9.6516	1.95	methenamine
7	10.8737	3.92	4-hydroxy-3-methylacetophenone
8	12.0275	1.64	vanillin
9	12.6653	1.51	*trans*-isoeugenol
10	13.0548	1.36	6-methoxy-3-methylbenzofuran
11	15.3139	1.69	4-((1E)-3-hydroxy-1-propenyl)-2-methoxyphenol (also named coniferol or γ-hydroxyisoeugenol)
12	15.5865	0.82	2-hydroxy-4-isopropyl-7-methoxytropone
13	15.9370	1.68	4-hydroxy-3-methoxybenzeneacetic acid, -, methyl ester
14	16.0636	45.64	4-((1E)-3-Hydroxy-1-propenyl)-2-methoxyphenol (also named coniferol or γ-hydroxyisoeugenol)
15	17.1153	14.99	2-propenoic acid, 3-(4-hydroxy-3-methoxyphenyl)-, methyl ester (also named ferulic acid methyl ester)
16	17.9234	0.49	ethyl (2E)-3-(4-hydroxy-3-methoxyphenyl)-2-propenoate
17	19.5447	0.67	9,15-octadecadienoic acid, methyl ester, (Z,Z)-
18	21.1027	2.93	2-(1,4,4-trimethyl-cyclohex-2-enyl)-ethanol
19	24.4377	2.13	9,12-octadecadienoic acid (Z,Z)-, 2,3-dihydroxypropyl ester

Peak = peak identification; Rt = retention time, expressed in minutes; Area = relative peak area percentage.

**Table 4:** Estimated EC_50_ and EC_90_ effective concentrations. Values are expressed in µg·mL^−1^, and are followed by the standard errors of the fit.

Pathogen	EC	Stevioside	*S. marianum*	Stevioside– *S. marianum*	Coniferyl Alcohol	Stevioside– Coniferyl Alcohol	Ferulic Acid	Stevioside– Ferulic Acid
*N. parvum*	EC_50_	152.2 ± 13.4	677.2 ± 47.0	89.2 ± 15.3	214.3 ± 26.2	157.8 ± 16.6	1394.5 ± 63.0	465.9 ± 27.51
	EC_90_	824.1 ± 56.7	2938.3 ± 286.6	262.1 ± 19.2	1005.1 ± 71.3	384.9 ± 22.8	2948.6 ± 268.1	1132.7 ± 127.3
*D. viticola*	EC_50_	271.4 ± 26.6	1088.4 ± 93.8	148.3 ± 11.7	361.1 ± 38.8	156.5 ± 8.3	1387.2 ± 134.3	544.5 ± 24.4
	EC_90_	1017.0 ± 74.3	9943.2 ± 1038.6	360.7 ± 39.0	988.5 ± 88.6	368.2 ± 26.6	3921.3 ± 438.6	1183.2 ± 111.0
*D. seriata*	EC_50_	230.1 ± 15.3	703.0 ± 26.6	127.1 ± 15.5	370.3 ± 10.4	191.6 ± 12.6	433.0 ± 31.5	209.0 ± 18.1
	EC_90_	840.5 ± 62.3	1461.1 ± 111.8	355.4 ± 38.1	913.2 ± 65.6	360.5 ± 29.6	903.4 ± 74.4	465.9 ± 33.2

*N. parvum* = *Neofusicoccum parvum*; *D. viticola* = *Dothiorella viticola*; *D. seriata* = *Diplodia seriata*; *S. marianum* = *Silybum marianum*; EC = effective concentration; EC_50_ and EC_90_ = 50% and 90% effective concentrations, respectively.

**Table 5:** Synergy factors for the stevioside–*S. marianum* conjugate complex against the three *Botryosphaeriaceae* taxa.

Effective Concentration	*N. parvum*	*D. viticola*	*D. seriata*
EC_50_	2.8	2.9	2.7
EC_90_	4.9	5.1	3.0

*N. parvum* = *Neofusicoccum parvum*; *D. viticola* = *Dothiorella viticola*; *D. seriata* = *Diplodia seriata*; *S. marianum* = *Silybum marianum*; EC_50_ and EC_90_ = 50% and 90% effective concentrations, respectively. Synergy factors are expressed as absolute values.

**Table 6:** Kruskal–Wallis test and multiple pairwise comparisons using the Conover–Iman procedure for the lengths of the vascular necroses for the three phytopathogen in greenhouse in vivo assays.

Pathogen	Sample	Frequency	Sum of Ranks	Mean of Ranks	Groups		
*N. parvum*	Stevioside–*S. marianum* negative control	48	1275.500	26.573	A		
	Stevioside–*S. marianum*	64	5911.000	92.359		B	
	Positive control	64	8389.500	131.086			C
*D. viticola*	Stevioside–*S. marianum* negative control	48	2174.000	45.292	A		
	Stevioside–*S. marianum*	64	4272.000	66.750		B	
	Positive control	64	9130.000	142.656			C
*D. seriata*	Stevioside–*S. marianum* negative control	48	2062.500	42.969	A		
	Stevioside–*S. marianum*	72	5641.500	78.354		B	
	Positive control	56	7872.000	140.571			C

*N. parvum* = *Neofusicoccum parvum*; *D. viticola* = *Dothiorella viticola*; *D. seriata* = *Diplodia seriata*; *S. marianum* = *Silybum marianum*. Treatments/controls labelled with the same letters are not significantly different at *p* < 0.05.

## Data Availability

The data presented in this study are available on request from the corresponding author. The data are not publicly available due to their relevance to be part of an ongoing Ph.D. Thesis.

## Supplementary Materials

The following are available online at https://www.mdpi.com/article/10.3390/plants10071363/s1, Table S1. Repetitions for each of the plant/treatment/pathogen combinations in the greenhouse bioassay; Table S2. Kruskal-Wallis test and multiple pairwise comparisons using the Conover-Iman procedure for the lengths of the vascular necroses in greenhouse in vivo assays considering two independent variables (treatment and taxa); Figure S1. Biosynthesis of silybins from taxifolin and coniferyl alcohol; Figure S2. Formation of coniferyl alcohol; Figure S3. Infrared spectrum of *S. marianum* extract (after lyophilization); Figure S4. GC–MS spectrum of *S. marianum* hydromethanolic extract; Figure S5. Chemical structures of some of the phytochemicals identified by GC-MS in the hydro-methanolic extract of *S. marianum*.
