# Supplementary material for: Antifungal Activity against Botryosphaeriaceae Fungi of the Hydro-Methanolic Extract of Silybum marianum Capitula Conjugated with Stevioside
Source: Plants (Basel). 2021 Jul 3;10(7):1363. doi: 10.3390/plants10071363 (PMC8309442; doi:10.3390/plants10071363)
Supplement: Supplementary file 1 [file plants-10-01363-s001.zip › plants-1284147-supplementary.pdf]

Antifungal Activity against *Botryosphaeriaceae* Fungi of the Hydro-Methanolic Extract of *Silybum marianum* Capitula Conjugated with Stevioside

Natalia Langa-Lomba, Laura Buzón-Durán, Eva Sánchez-Hernández, Pablo Martín-Ramos, José Casanova-Gascón, Jesús Martín-Gil, Vicente González-García

SUPPORTING INFORMATION

Table S1. Repetitions for each of the plant/treatment/pathogen combinations in the greenhouse bioassay. Each grafted plant was artificially inoculated at two sites below grafting point.

Plant	Treatment	Pathogen	Number of replicates
'Tempranillo' (CL. 32 clone) on 775P rootstock	Stevioside–*S. marianum*	*N. parvum*	5
		D. viticola	5
		D. seriata	5
		None (negative control)	3
	None (positive control)	*N. parvum*	4
		D. viticola	4
		D. seriata	4
'Garnacha' (VCR3 clone) on 110R rootstock	Stevioside–*S. marianum*	*N. parvum*	5
		D. viticola	5
		D. seriata	5
		None (negative control)	3
	None (positive control)	*N. parvum*	4
		D. viticola	4
		D. seriata	4

Table S2. Kruskal–Wallis test and multiple pairwise comparisons using the Conover–Iman procedure for the lengths of the vascular necroses in greenhouse *in vivo* assays considering two independent variables (treatment and taxa).

Sample	Frequency	Sum of ranks	Mean of ranks	Groups			
Stevioside–*S. marianum* negative control	48	3160.000	65.833	A			
D. viticola|Stevioside–*S. marianum*	64	6628.000	103.563	A	B		
D. seriata|Stevioside–*S. marianum*	72	9602.000	133.361		B		
N. parvum|Stevioside–*S. marianum*	64	15532.000	242.688			C	
D. seriata|Positive control	56	16569.500	295.884				D
D. viticola|Positive control	64	20520.000	320.625				D
N. parvum|Positive control	64	21516.500	336.195				D

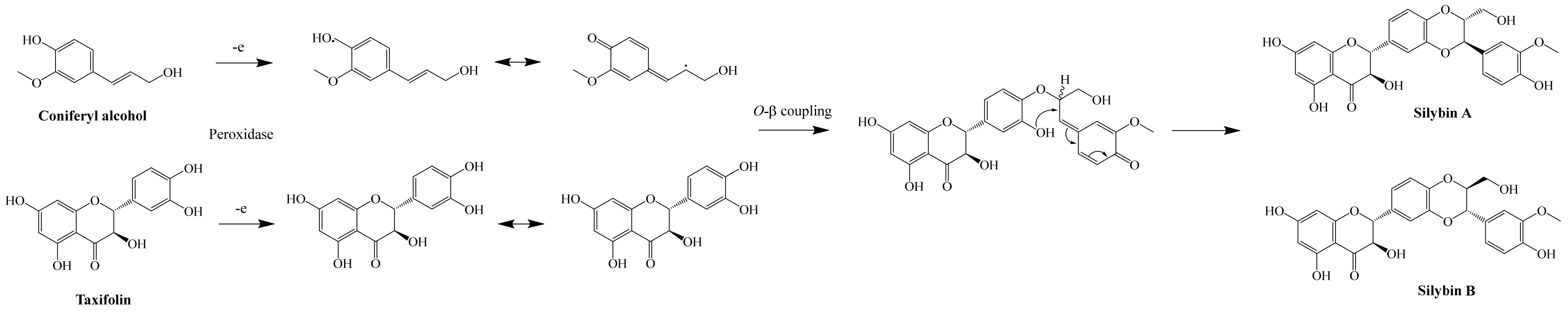

Figure S1. Biosynthesis pathway of silybins from taxifolin and coniferyl alcohol.

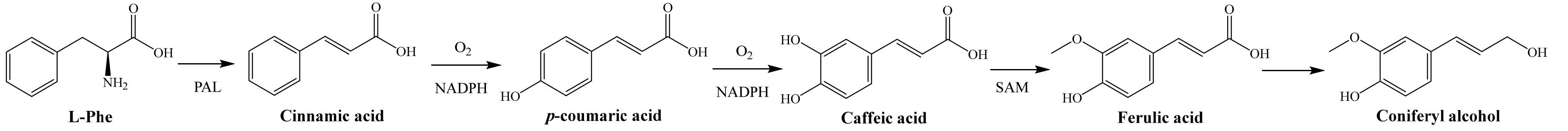

Figure S2. Formation of coniferyl alcohol.

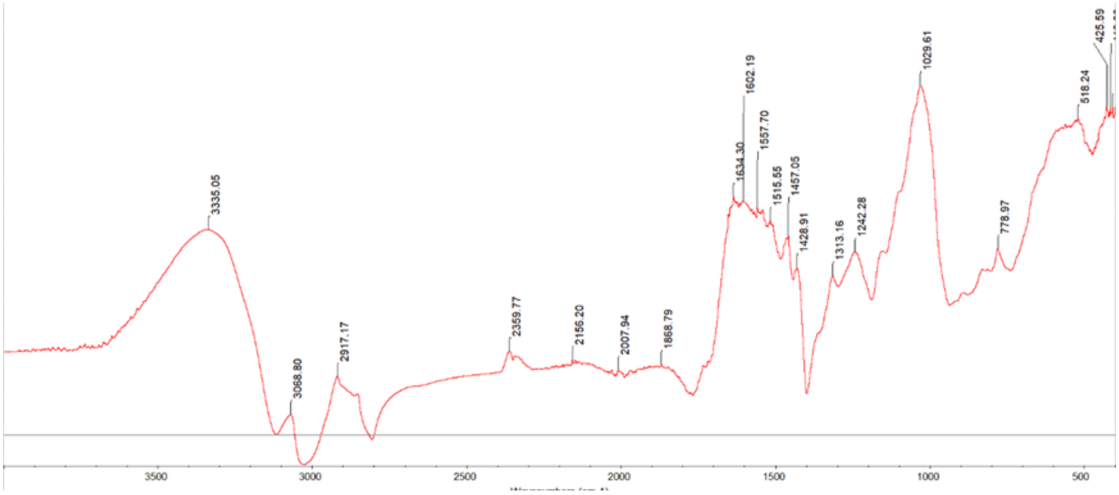

Figure S3. Infrared spectrum of *S. marianum* extract (after lyophilization).

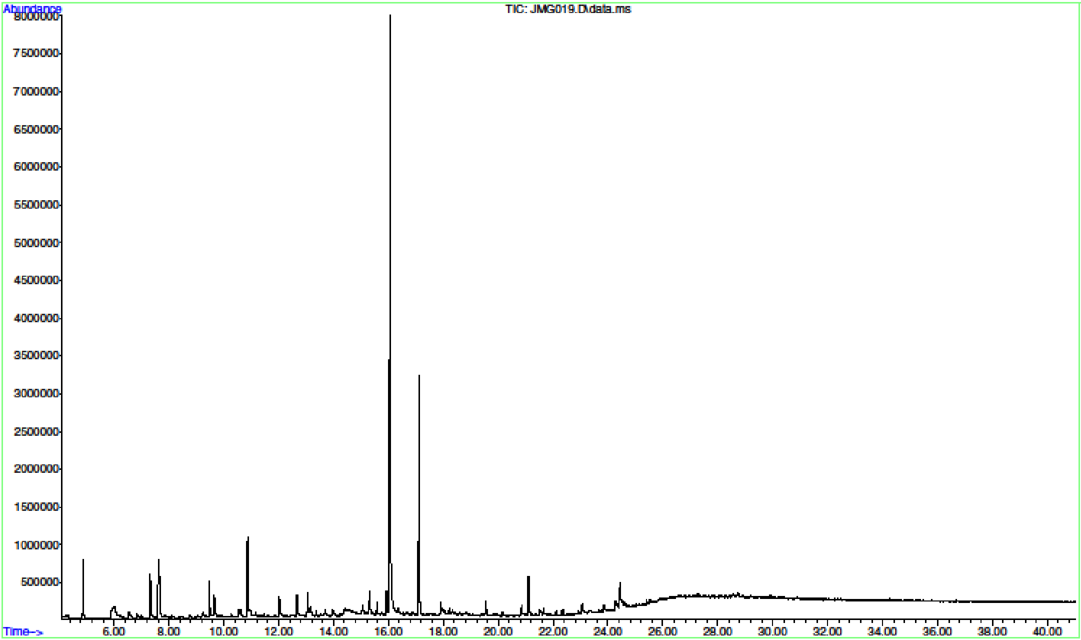

Figure S4. GC–MS spectrum of *S. marianum* hydromethanolic extract.

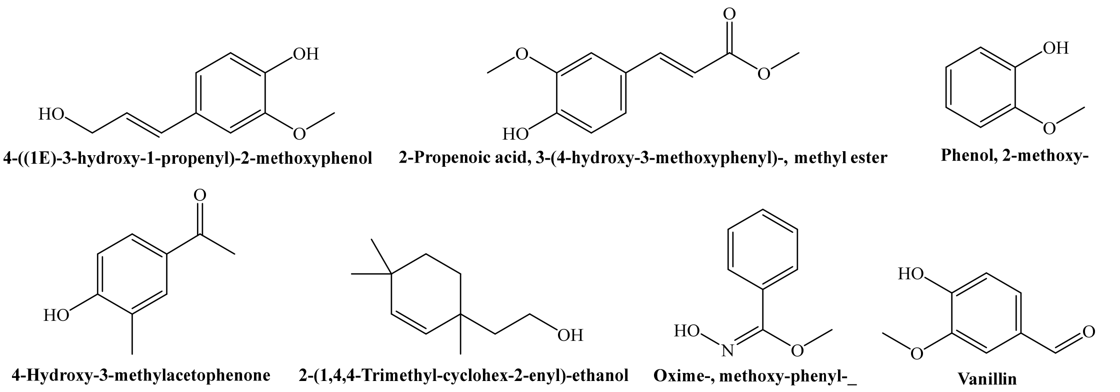

Figure S5. Chemical structures of some of the phytochemicals identified by GC-MS in the hydro-methanolic extract of *S. marianum.*
